# What are the applications of single-cell RNA sequencing in cancer research: a systematic review

**DOI:** 10.1186/s13046-021-01955-1

**Published:** 2021-05-11

**Authors:** Lvyuan Li, Fang Xiong, Yumin Wang, Shanshan Zhang, Zhaojian Gong, Xiayu Li, Yi He, Lei Shi, Fuyan Wang, Qianjin Liao, Bo Xiang, Ming Zhou, Xiaoling Li, Yong Li, Guiyuan Li, Zhaoyang Zeng, Wei Xiong, Can Guo

**Affiliations:** 1 NHC Key Laboratory of Carcinogenesis and Hunan Key Laboratory of Cancer Metabolism, Hunan Cancer Hospital and the Affiliated Cancer Hospital of Xiangya School of Medicine, Central South University, Changsha, China; 2 Key Laboratory of Carcinogenesis and Cancer Invasion of the Chinese Ministry of Education, Cancer Research Institute, Central South University, Changsha, China; 3 Department of Stomatology, Xiangya Hospital, Central South University, Changsha, China; 4 Department of Oral and Maxillofacial Surgery, The Second Xiangya Hospital, Central South University, Changsha, China; 5 Hunan Key Laboratory of Nonresolving Inflammation and Cancer, Disease Genome Research Center, The Third Xiangya Hospital, Central South University, Changsha, China; 6 Department of Medicine, Dan L Duncan Comprehensive Cancer Center, Baylor College of Medicine, Houston, TX USA

**Keywords:** Single-cell RNA sequencing, Sequencing platform, Tumor, Specimen preparation, Data analysis

## Abstract

Single-cell RNA sequencing (scRNA-seq) is a tool for studying gene expression at the single-cell level that has been widely used due to its unprecedented high resolution. In the present review, we outline the preparation process and sequencing platforms for the scRNA-seq analysis of solid tumor specimens and discuss the main steps and methods used during data analysis, including quality control, batch-effect correction, normalization, cell cycle phase assignment, clustering, cell trajectory and pseudo-time reconstruction, differential expression analysis and gene set enrichment analysis, as well as gene regulatory network inference. Traditional bulk RNA sequencing does not address the heterogeneity within and between tumors, and since the development of the first scRNA-seq technique, this approach has been widely used in cancer research to better understand cancer cell biology and pathogenetic mechanisms. ScRNA-seq has been of great significance for the development of targeted therapy and immunotherapy. In the second part of this review, we focus on the application of scRNA-seq in solid tumors, and summarize the findings and achievements in tumor research afforded by its use. ScRNA-seq holds promise for improving our understanding of the molecular characteristics of cancer, and potentially contributing to improved diagnosis, prognosis, and therapeutics.

**Supplementary Information:**

The online version contains supplementary material available at 10.1186/s13046-021-01955-1.

## Background

Tumors are generally considered to be of monoclonal origin, with mutations facilitating the expansion of a malignant cell in the body to visible tumor tissue, as well as from carcinoma in situ to metastatic carcinoma. Notably, patient-derived tumor tissue includes cancer cells as well as other cell types, such as infiltrating immune cells and fibroblasts. Therefore, the use of traditional bulk RNA sequencing technology can only permit an average qualitative characterization of such highly complex tissue, representing the average of cancer cells and non-cancer cells. Although bulk RNA approaches have made many invaluable contributions to medical science [1], such approaches have ignored the distinct phenotypical and functional traits of single cells within tumor samples.

Since the first single-cell RNA sequencing (scRNA-seq) study was published in 2009 [2], various commercial platforms and methods have been developed for scRNA-seq. ScRNA-seq is a technique allowing for the study of tissues at single-cell resolution. Many researchers within the life sciences employ scRNA-seq for the investigation of diverse biological functions. In particular, great achievements have been made in tumor research using scRNA-seq technology. The single-cell resolution afforded by scRNA-seq enables direct measurement of the transcriptional output of cells from tumor samples [3], comparison of differences between the transcriptomes of various cells, identification of rare cell subpopulations, such as heterogeneous tumor subpopulations [4], or the revelation of differences between stimulated dendritic cells [5], in turn providing unprecedented insights that have contributed to the development of cancer therapy.

In the present review, we introduce methods for the preparation of solid tumor samples, related scRNA-seq platforms, the analysis of scRNA-seq data, and achievements in tumor research facilitated by the use of scRNA-seq.

## Single-cell RNA sequencing

To carry out scRNA-seq, solid tumor samples first need to be processed to effectively isolate viable single cells from the tissue of interest [6]. Thereafter, the single cells are lysed to obtain RNA, which is reverse transcribed into cDNA and then amplified to construct a sequencing library. The most suitable sequencing instrument must be selected based on the experimental scheme and research objectives. After sequencing, the data need to be correctly analyzed to reveal new findings.

### Preparation of solid tumor specimens

Many published articles on the use of scRNA-seq in cancer research have detailed the preparation of solid tumor specimens. After cancer is diagnosed, tumor tissues are removed by biopsy and treated immediately to preserve single cells. Tumor tissue is usually cut into sections of approximately 1 mm^3^ and washed with PBS to remove fat, visible vessels, and surrounding necrotic areas [7]. Tissue is separated into single cells via grinding and filtration [8], using a Human Tumor Dissociation Kit [7, 9], or through other methods. Separated cells are then centrifuged and resuspended. All samples are stained with trypan blue to confirm viability, evaluate sample quality, and remove dead cells. To avoid introducing gene expression changes associated with processing, after washing and counting, cell suspensions are preserved in cold storage in preparation for cell sorting.

### Selection of sequencing platform

Currently, major single-cell sequencing platforms include the 10X Genomics Chromium, Nadia (Dolomite Bio), Illumina Bio-Rad ddSEQ Single-Cell Isolator, BD Rhapsody Single-Cell Analysis System (BD), ICELL8 Single-cell System (Takara), Fluidigm C1, and others. Although new platforms for scRNA-seq are still being developed, the most widely used platform remains the 10X Genomics Chromium.

Selecting the most suitable sequencing platform for your research project is a key step for achieving the desired research results. There are a number of published articles comparing performance, cost, and other aspects of existing platforms. In 2019, Zhang et al. [10] compared three widely used drop-based high-throughput scRNA-seq systems: inDrop, Drop-seq, and 10X Genomics Chromium, and found that the 10X Genomics Chromium had the highest sensitivity and the lowest technical noise. The 10X Genomics platform is therefore a more suitable choice for rare samples, such as human embryos, which require a more efficient cell capture platform. For abundant samples, Drop-seq may be more cost-effective, while InDrop is a better choice when a customized protocol is necessary. Natarajan et al. [11] compared the BGISEQ-500 platform with Illumina HiSeq and observed that these platforms were comparable with regard to sensitivity, accuracy, and repeatability. However, the sequencing cost of the former was lower. These comparative studies indicate that each sequencing platform has its own merits, and no sequencing platform is suitable for all research objectives. In summary, researches have to choose the sequencing platform that can best meet their specific experimental needs.

### Analysis of scRNA-seq data

Data generated during scRNA-seq usually gets processed via two analytical procedures: pre-processing (including quality control, batch-effect correction, and normalization) and downstream analysis (cell cycle phase assignment, clustering, reconstruction of cell trajectory and pseudo time, differential expression and gene set enrichment analysis, as well as gene regulatory network inference). The following section will briefly introduce the significance of and methods for these analytical procedures.

### Quality control

Quality control should always be carried out as the first step for scRNA-seq data. Various methods for quality control have been developed [12–14]. Data obtained from poor quality cells (poor activity, containing degraded RNA, and zero expression levels of housekeeping genes such as GAPDH and ACTB) [6] should be excluded before subsequent bioinformatics analysis to mitigate its influence on downstream analysis results. The percentage of mitochondrial reads is a common quality control metric [15]. When there are a large number of mitochondrial transcripts, it means that the cells are in a state of stress [16], so a threshold is commonly applied to exclude data from cells with too many mitochondrial transcripts. Similarly, the proportion of ribosomal reads is another commonly used quality control metric. Because scRNA-seq is mainly used to study functional (messenger) RNA, cells that have had their ribosomes removed and still have a high proportion of ribosome reads cannot be further analyzed [17]. In addition to the quality control methods for a single dataset, a method denoted ‘scRNABatchQC’ has been proposed that facilitates quality assessment across datasets to intuitively detect biases and outliers [18]. To aid researchers who are intimidated by scRNA-seq analysis, Etherington et al. [19] developed tools and training materials that can be used for scRNA-seq training and quality control.

### Batch-effect correction

During the scRNA-seq experimental procedure, when cells subject to different conditions are cultured, captured, and sequenced separately, batch effects will be evident [20]. There are several methods available for batch-effect correction of scRNA-seq data, including Seurat 3 [21], MMD-ResNet [22], Harmony [23], Scanorama [24], Liger [25], scMerge [26], ZINB-WaVE [27], and others. Based on a variety of evaluation indicators, Harmony, Liger, and Seurat 3 are the recommended methods for dealing with batch effects, among which Harmony is the first choice due to its shorter run time [28]. Recently, a novel numerical algorithm for batch-effect correction of bulk and scRNA-seq data was proposed, denoted ‘scBatch’. This approach is not limited by the hypothesis of the batch-effect generation mechanism, and is superior to the benchmark batch-effect correction algorithms [29].

### Normalization

Data normalization is essential for scRNA-seq to make gene expression comparable within and/or between samples. A number of methods have been developed for the normalization of RNA-seq data [30–33]. However, the majority of methods follow the same principle as bulk RNA-seq normalization and, thus, are not applicable to scRNA-seq data [30, 31]. Nevertheless, several methods have recently been devised to normalize scRNA-seq data, such as SCONE [34] and regularized negative binomial regression [35]. SCONE provides a flexible framework for users to choose appropriate normalization methods. Normalization using regularized negative binomial regression effectively eliminates technical differences due to different sequencing depth without inhibiting biological heterogeneity. A previous study [36] compared seven scRNA-seq data normalization methods with regard to reduction of noise or bias, and found that each of these methods was suitable to normalize specific types of data for further downstream analysis.

### Cell cycle phase assignment

Determining the cell cycle phase of a single cell can facilitate understanding of biological processes such as tumorigenesis [37–41] and cell differentiation [42, 43], and avoid the confounding effects caused by the cell cycle phase prior to downstream analysis. Scialdone et al. [44] described and compared six supervised cell cycle prediction methods based on a cell transcriptome, of which the parameter-free PCA-based method and the custom predictor known as the “Pairs” method performed best in allocating cells to the correct cell cycle stage. Buettner et al. [45] proposed a calculation method, denoted ‘single-cell latent variable model’ (scLVM), which can be used to eliminate variations caused by cell cycle and other confounding factors before downstream analysis. Recently, Hsiao et al. [46] proposed a new method to characterize the progress of the cell cycle, which is different from the traditional classification of cells according to the standard of cell cycle stage (G1, S or G2/M phase), but can quantify the cell cycle progression of induced pluripotent stem cells on a continuum, which provides a basis for the characterization of the cell cycle in other cell types.

### Cell clustering

One of the basic goals of scRNA-seq data analysis is to identify cell types from experimental samples to elucidate tissue complexity and heterogeneity. Due to the importance of cell type recognition, efforts have been made to develop new algorithms, including CountClust [47], CIDR [48], SIMLR [49], SAFE [50], and other advanced methods. A few studies [51–54] have compared and summarized diverse clustering algorithms for scRNA-seq data analysis. Unlike previous methods, Geddes et al. [55] proposed the first ensemble clustering framework based on autoencoder dimension reduction, which could be combined with different clustering algorithms to promote the accurate recognition of cell types. Since then, several new clustering methods have emerged, including DivBiclust [56], a biclustering-based framework, SAME [57], which extracts cluster solutions from multiple methods, and PARC [58], which is suitable for large-scale single-cell data. To overcome flaws associated with the manual labeling of cell types, Shao et al. [59] developed an automatic annotation toolkit, denoted ‘scCATCH’, based on clustering, which can accurately annotate cell types with acceptable repeatability.

### Reconstruction of cell trajectory and pseudo-time

By reconstructing cell trajectory and pseudo-time based on scRNA-seq data, dynamic processes in cells can be calculated and simulated, which is of great significance for understanding the transition between cell states in cancer [60]. Currently available algorithms include Monocle 2 [61], Monocle 3 [62], TSCAN [63], Slingshot [64], SLICE [65], LISA [66], p-Creode [67], Waddington-OT [68] and others. Considering the exponential growth in the size of scRNA-seq data, Chen et al. [66] proposed an unsupervised method, denoted ‘Lisa’, for the reconstruction of cell trajectory and pseudo-time for a large number of scRNA-seq datasets. p-Creode is another unsupervised algorithm that can predict cell state-transition trajectories. Waddington-OT uses the mathematical method of optimal transport (OT) to infer ancestor-descendant fate, and reconstruct cell trajectories. In addition to the three methods mentioned above, the algorithms Monocle 2 (the new version Monocle 3), TSCAN, and Slingshot have been shown to have good performance at reconstructing cell trajectory and presudo-time [69].

### Differential expression and gene set enrichment analysis

One of the most common uses of gene expression data is for the identification of differentially expressed (DE) genes under different experimental conditions (e.g., stimulated versus non-stimulated, mutant versus wild-type, or between different time points), and thus to determine the root cause of phenotypic differences observed under different conditions [70]. A zero value for a gene’s expression level in scRNA-seq data may indicate two things. One is the “real” zero, caused by the changing characteristics of single-cell gene transcription, while the other is the “dropout” zero, caused by technical reasons, which often affects the validity of differential expression analysis. Miao et al. [71] developed the R package DESingle, which can accurately distinguish between the two types of zeros. DECENT is a DE gene analysis method based on UMI scRNA-seq data, and is used to analyze the pre-dropout distributions of inferred RNA molecules [72]. In addition to dropout zeros, another challenge in differential expression analysis of scRNA-seq data is multimodal data distribution. ZIAQ is the first approach to consider both dropout rates and the complex distributions of scRNA-seq data, which can be used to identify more DE genes [73].

We usually group DE genes according to their participation in common biological processes to facilitate the interpretation of results [74]. Existing gene set enrichment (GSE) analysis methods include DAVID [75], PAGE [76], CAMERA [77] and others, but almost all of these methods are more suitable for bulk RNA-seq analysis [78]. In addition, almost all existing GSE methods are used as a separate step after DE analysis. Considering the above shortcomings, Ma et al. [79] proposed IDEA, a computational method integrating DE analysis and GSE analysis for scRNA-seq, which could greatly improve the outcomes of both.

### Gene regulatory network inference

The combination of active transcription factors and their target genes is usually described in gene regulatory networks (GRNs). Revealing these regulatory interactions is the goal of GRN inference methods, providing valuable insights for the identification of causal regulatory factors in biological processes [74]. A class of GRN inference methods are based on Boolean network models, such as SCNS toolkit [80] and BTR [81]. Another approach for the inference of regulatory networks is based on co-expression analysis, and example models include SINCERA [82], which is specifically used for scRNA-seq data. In addition, there are algorithms based on ordinary differential equations, such as SCODE [83] and InferenceSnapshot [84]. Recently, Moerman et al. [85] proposed the GRNBoost2 and Arboreto frameworks, which can help researchers to deduce high-quality GRNs from large datasets in a reasonable amount of time.

## Progress of single-cell RNA sequencing in tumors

Cancer patients may be unresponsive to therapy due to drug resistance and metastasis of single cells, both of which constitute major challenges in the treatment of malignant tumors. About 90% of available drugs are effective in less than half of patients [86]. Cancer is associated with the interaction of thousands of gene products, and genotype as well as interactions vary greatly within and between tumors [87], which is a key reason for the failure of some drugs. In contrast to bulk analysis, which does not account for the differences between cancer cells and their cancer-related counterparts, scRNA-seq provides unprecedented high resolution for the analysis of each individual malignant cell, stromal cell, endothelial cell, parenchymal cell, and immune cell, as well gene expression and pathway activation [88]. Thus, scRNA-seq provides insights that contribute to the development of strategies for cancer treatment and personalized medicine (see Additional file 1).

### Highlighting intra- and inter-tumoral heterogeneity

The considerable heterogeneity of tumors and tumor tissue samples between different patients is an important reason for treatment failure [89]. Therefore, understanding the functional status of individual tumor cells and recognizing cell subset composition and characteristics is of great significance for cancer biology and treatment strategies (Table 1 (see Additional file 2)).

Glioblastoma (GBM) is the most common primary malignant brain tumor in adults [90]. It is the glioma with the highest degree of malignancy, and the most often seen in clinical practice, with poor prognosis and a lack of effective treatment regimens [91]. In 2014, Patel et al. [92] analyzed 430 cells of 5 primary GBMs (all IDH1/2 wild-type primary GBMs) using scRNA-seq, and found that these cells differed in the expression of various programs related to carcinogenic signaling pathways, cell proliferation, the immune response, and hypoxic stress. In 2018, Yuan et al. [93] analyzed high-grade glioma (HGG) with large-scale parallel scRNA-seq using a high-density microwell system, and found that, similar to oligodendrocyte progenitors, glioma cells exhibited proliferative characteristics. In contrast, similar to astrocytes, neuroblasts, and oligodendrocytes, glioma cells exhibited an amitotic state in tumors.

Melanoma is a highly malignant skin cancer with four clinically distinguishable subtypes and is responsible for approximately half of skin cancer-related deaths in Japan [94]. Gerber et al. [95] used scRNA-seq to analyze transcription in cells from three different metastatic melanoma patients (BRAF/NRAS wild type, BRAF mutant/NRAS wild type, and BRAF wild type /NRAS mutant). BRAF/NRAS wild-type samples had a low-abundance subgroup with high expression of ABC transporters, while cells from the other two samples exhibited more homogeneous single-cell gene expression patterns.

Head and neck squamous cell carcinoma (HNSCC) encompasses a group of malignant tumors originating from the squamous epithelium of the oral cavity, oropharynx, larynx, and hypopharynx [96–98]. Puram et al. [9] generated scRNA-seq profiles of HNSCC in 18 patients and found that stromal and immune cells had the same expression program per patient, whereas the expression programs of malignant cells within and between tumors were different with regard to cell cycle, hypoxia, stress, and epithelial differentiation.

Lung cancer (LC) can be divided into two subtypes: small-cell lung carcinoma (SCLC) and non-small-cell lung carcinoma (NSCLC), among which NSCLC accounts for the majority of LC cases [99]. Lambrechts et al. [100] analyzed 92,948 NSCLC cells (84,341 stromal cells) at single-cell resolution, and identified 52 subtypes of stromal cells, providing a comprehensive list of stromal cell types. Based on these findings, the tumor microenvironment (TME) of lung cancer is more complex and heterogeneous than previously thought.

Acute myeloid leukemia (AML) is a highly heterogeneous hematological malignancy. Van Galen et al. [101] profiled the cells of 16 patients with AML and 5 healthy donors by combining scRNA-seq with single-cell genotyping, and then correctly classified the cell types by machine learning, which could correctly distinguish normal cells from cancer cells, and identified 6 malignant cell types, which comprehensively parsed the heterogeneous ecosystem of AML.

### Discovery of invasion and metastasis mechanisms

The ability of single-cell gene expression profiling to identify specific patterns of gene expression allows for the elucidation of mechanisms underlying tumor invasion and metastasis [102–105] that are critical for preventing the spread of cancer, which considerably complicates treatment (Table 2 (see Additional file 3)).

Pancreatic cancer is one of the leading causes of cancer-related death in developed countries, and it ranks 7th in cancer-related deaths among both men and women [106]. Ting et al. [107] obtained high-quality transcripts from 93 single mouse pancreatic circulating tumor cells (CTCs) and compared them with scRNA-seq data from pancreatic cancer patients. The expression of an extracellular matrix protein-encoding gene, SPARC, was abnormally high in both mouse and human pancreatic CTCs, and its knockdown decreased the ability of cancer cells to invade and metastasize, indicating that the gene is closely related to pancreatic cancer invasion and metastasis. In 2017, Puram et al. [9] identified partial epithelial-to-mesenchymal transition (p-EMT) in malignant HNSCC tumor cells through scRNA-seq. Cells exhibiting p-EMT activation were located at the outer area of primary tumors, promoting the invasion and metastasis of tumor cells.

Breast cancer is by far the most common malignancy in women [108] as well as the leading cause of cancer-related death in women [109]. Chung et al. [110] conducted transcriptomic analysis of 515 single cells from patients with different breast cancer subtypes, revealing the gene expression patterns of cancer cells and immune cells. It is worth noting that rare cell types exhibiting pronounced EMT and stemness phenotypes were identified in triple-negative breast cancer (TNBC), and these may be a driving force of tumor progression and metastasis. Multiple myeloma (MM) is the second most common hematologic malignancy and cannot be completely cured [111]. Geng et al. [112] performed single-cell transcriptomic analysis of cells taken from 21 MM patients, and found that the chemokine CXCL12 was abnormally upregulated in circulating plasma cells (cPCs). CXCL12 may prompt cPCs to escape bone marrow retention and migrate into the bloodstream, eventually forming an extramedullary plasmacytoma (EMP).

### Study of drug resistance mechanisms

When anti-cancer drugs are used to target tumor cells, some cells develop resistance to drugs and contribute to disease relapse, rendering treatment ineffective. ScRNA-seq can be employed to distinguish between primary and recurrent tumor cells, providing insight into the cell subsets responsible for tumor recurrence, mutated genes, and multiple pathways that drive tumor growth. Thus, scRNA-seq can guide strategies for the treatment of tumors (Table 3 (see Additional file 4)).

In 2014, Lee et al. [113] employed scRNA-seq in metastatic breast cancer cells treated with the chemotherapeutic agent paclitaxel. A small number of cells failed to respond to paclitaxel by producing specific RNA variants, but these transcripts were not detected in the two groups of cells that were not treated with chemotherapy or effective chemotherapy. In 2015, Kim et al. [114] conducted scRNA-seq on 34 patient-derived xenograft (PDX) cells isolated from a xenograft tumor of a lung adenocarcinoma (LUAD) patient, and identified a tumor cell subpopulation related to anti-cancer drug resistance that expressed KRAS^G12D^ through gene expression and mutation profiling. In 2016, Tirosh et al. [4] performed scRNA-seq on 4645 cells isolated from 19 patients with melanoma, and found that all tumors contained cells with two transcriptional states, namely “MITF-high” and “AXL-high”. After treatment with RAF/MEK inhibitors, the number of “AXL-high” cells gradually increased from their initially small population. However, the proportion of “AXL-high” cells in a lot of the tumors remained almost unchanged, indicating that the cells with high AXL expression were related to targeted therapy resistance. In 2018, Chen et al. [115] performed scRNA-seq in over 200 single cells (from patients with primary and relapsed glioblastoma multiforme), revealing relapse pathways in GBM. A trio of mutated genes, all involved in the RAS/GEF GTP-dependent signaling pathway, were identified in single cells from relapsed GBM multiforme, but not in those from primary tumors. The authors further confirmed the identified molecular pathway by meta-analysis of RNA-seq data from thousands of patients.

Medulloblastoma (MB) is one of the most common malignant brain tumors in children, with a higher incidence in children aged 3 to 4 years and 8 to 10 years, and is slightly more prevalent in boys [116], with no significant racial bias [117]. In 2019, Ocasio et al. [118] analyzed the response of Sonic Hedgehog (SHH)-driven mouse MB to the SHH-pathway inhibitor vismodegib by using scRNA-seq and lineage tracking. Vismodegib could reduce some proliferative cell subgroups and promote the differentiation of certain cells. However, specific cell types continued to proliferate in vismodegib-treated tumors, exhibiting either sustained SHH-pathway activation or stem cell characteristics.

### Characterization of the tumor immune microenvironment

Various types of immune cells infiltrate tumor tissue [119–128]. The gene expression status of each myeloid cell and lymphocyte can be obtained via scRNA-seq, providing insights into the immune status of cancers. Such scRNA-seq data is a valuable asset for formulating effective immunotherapy approaches for cancer, alongside studying the basic characteristics of tumor-infiltrating immune cells (Table 4 (see Additional file 5)).

Hepatocellular carcinoma (HCC) usually occurs in the context of advanced chronic liver disease, and is primarily associated with hepatitis B or C virus infection and alcohol abuse, accounting for 70–85% of the total cases of liver cancer [129]. Zheng et al. [8] performed deep scRNA-seq on 5,063 T cells isolated from tumors, peripheral blood, and the adjacent normal tissue of 6 HCC patients. They observed that 11 T cell subsets were present in the HCC microenvironment. The gene *LYAN* was highly expressed in activated CD8+ T cell and Tregs, which could inhibit the function of CD8+ T cells. In 2017, Muller et al. [130] applied scRNA-seq to glioblastoma-derived myeloid cells, particularly tumor-associated macrophages (TAMs), for the first time. The results revealed that blood-derived TAMs and microglial TAMs exhibited different phenotypes and localization within the tumor. Compared with microglial TAMs, blood-derived TAMs could upregulate immunosuppressive cytokines, exhibited metabolic changes, and were enriched in perivascular and necrotic areas. Moroever, in 2017, researchers from another group [131] used paired single-cell analysis to study the innate immunity of early-stage LUAD, and found a large number of Treg and non-functional T cells, as well as NK cell depletion, in a stage I tumor, indicative of compromised anti-tumor immunity. Some of the immune features found in advanced LC were also present in early LC. For example, PD-1 was present on some of the CD4+ and CD8+ T cells at both stages. In 2018, Azizi et al. [132] performed scRNA-seq on 45,000 immune cells, mapped the various immune cell phenotypes in the breast TME, and identified most of the immune cell types in 8 patients. Although the expression profiles of immune cells were quite similar between tumor and normal tissue samples, specific phenotypic expansion was observed in tumor immune cells, and T cells were in a continuous state of activation, which did not conform to the traditional polarization distribution model of cancer macrophages.

Colorectal cancer (CRC) is a common gastrointestinal malignancy, and is the third most common cancer in the United States [133]. Zhang et al. [134] obtained the transcriptomes of 11,138 single T cells from 12 patients with CRC by scRNA-seq, and quantitatively analyzed the dynamic relationship between the development, function, and migration of 20 T cell subsets through TCR tracking.

### Resolution of long disputed questions

ScRNA-seq can solve problems that previous technologies could not address, thus resolving debates and contributing to the development of oncology.

The relationship between human cytomegalovirus (HCMV) and GBM is an ongoing debate, as the detection of HCMV in GBM samples via molecular assays may be influenced by cellular heterogeneity. In 2017, Johnson et al. [135] aligned the scRNA-seq reads from 5 GBM tumors and 2 cell lines to HCMV, and found that no complete transcripts of the HCMV virus were discovered in either tumor samples or cell lines.

### Understanding the origin and evolution of tumors

ScRNA-seq can be used to better understand the occurrence and development of tumors at the cellular level, which enables to the exploration of new therapeutic methods (Table 5 (see Additional file 6)).

In 2016, Tirosh et al. [136] selected 4347 cells from 6 untreated grade II oligodendrogliomas (harboring IDH1 or IDH2 mutations) and analyzed them using scRNA-seq, and found that the cells with proliferative characteristics were highly enriched in a small number of undifferentiated rare subpopulations, suggesting that malignant proliferative cells within oligodendrogliomas could mainly originate from such a subgroup. In 2017, Venteicher et al. [137] combined scRNA-seq profiles from astrocytoma (IDH-A) and oligodendroglioma (IDH-O) tumors with 165 TCGA bulk RNA-seq profiles to analyze their genotype, phenotype, and the TME. The shared glial lineages and developmental hierarchy observed in both IDH-A and IDH-O tumors indicated that all IDH mutant gliomas had a common progenitor. In 2019, Saurty-Seerunghen et al. [138] analyzed public scRNA-seq data from surgically resected GBM samples and obtained a comprehensive view of metabolic pathways. ELOVL2 was closely related to the tumorigenicity of GBM cells, and knockdown of the ELOVL2 gene could inhibit GBM tumorigenicity, indicating that the overexpression of ELOVL2 promoted the growth of GBM. The authors further explored the mechanisms of ELOVL2 and found that the formation and release of extracellular vesicles is one of the pathways through which the regulation of which ELOVL2 controls GBM development.

In 2019, Hovestadt et al. [139] obtained 25 MB samples, including all molecular subgroups, and created the first cell map of all MB subgroups, revealing that group 4-MB probably originated from unipolar brush cells and glutamatergic cerebellar nuclei. In the same year, Weng et al. [140] identified Zfp36l1 as a key regulator of oligodendrocyte-astrocyte lineage transition and gliomagenesis by lineage-targeted single-cell transcriptomics analysis. Through experiments in mouse and human glioma cells, Zfp36l1 was demonstrated to control the genesis and growth of gliomas. Furthermore, gliomas with higher expression of Zfp36l1 were more difficult to treat. In 2020, Chen et al. [141] collected a large cohort of histone H3.3 G34R/V (glycine 34 to arginine or valine) HGGs (*n* = 95), and proved that G34R/V HGGs originated from interneuron progenitors expressing GSX2/DLX using scRNA-seq, and then confirmed this result using epigenomic profiles.

### Applications in anticancer research design

At present, practice has proven the guiding significance of scRNA-seq in anticancer protocol design, with important clinical application value in personalized diagnosis, treatment, and prognosis, as well as the evaluation of treatment effect for highly heterogeneous tumors (Table 6 (see Additional file 7)).

About 30% of patients with renal cell carcinoma (RCC) are diagnosed with metastases, and metastatic renal cell carcinoma (MRCC) is one of the most drug-resistant malignancies [142]. In 2016, Kim et al. [143] used scRNA-seq to detect the activation of targeted pathways in patients with refractory MRCC at the single-cell level. On the basis of predicting the activation of multiple drug target pathways, the authors proposed a combinatorial therapeutic strategy for the treatment of metastatic cancer. Whether in vitro or in vivo, the effect of this combined strategy was significantly improved compared to monotherapy. In 2019, Zhang et al. [144] proposed a multilayer network biomarker (MNB) based on the scRNA-seq data of IDH-mutant astrocytoma samples. MNB links cancer cells and the TME, allowing for prognosis and prediction of the therapeutic response in glioma patients.

### Identification of potential therapeutic targets

Modern cancer treatment approaches are often limited in their efficacy. In-depth analysis of malignant proliferative cells and immune cells at single-cell resolution is conducive to the identification of potential therapeutic targets, contributing to the development of new drug therapy and the improvement of patient survival rate (Table 7 (see Additional file 8)).

In 2017, Darmanis et al. [145] performed scRNA-seq on 3589 cells taken from the tumor core and surrounding tissue of 4 GBM patients. Groups of genes involved in size regulation, energy production, inhibition of apoptosis, regulation of intercellular adhesion, and central nervous system development were identified by analyzing the upregulated genes in infiltrating tumor cells, which could open up new avenues for treatment. In 2018, a study [146] conducted scRNA-seq on 3321 cells from 6 primary gliomas with histone H3 lysine27-to-methionine mutations (H3K27m-glioma) and matched models (PDX, gliomaspheres, and differentiated glioma cells), and found that H3K27m-glioma were mainly composed of cells similar to oligodendrocyte precursor cells (OPC-like). OPC-like cells exhibited greater proliferative ability and are maintained, at least in part, via PDGFRA signaling. In 2019, Wang et al. [147] extracted 16,128 tumor cells from epidermal growth factor receptor (EGFR) and EGFRvIII GBM tumors for scRNA-seq. RAD51AP1 was for the first time identified as an oncogene in GBM, highly related to EGFRvIII. This study revealed a new possibility for treatment using temozolomide combined with RAD51AP1, which could enhance the therapeutic effect and prolong patient survival.

Nasopharyngeal carcinoma (NPC) is an epithelial carcinoma arising from the lining of nasopharyngeal mucosa [148–152]. Although there is considerable NPC incidence in east and southeast Asia, it is not common compared to other types of cancer. Zhao et al. [7] constructed the first transcriptome profiles of primary NPC malignant cells and infiltrating immune cells using scRNA-seq. A high proportion of B cells infiltrating into the TMEs of three NPC patients was identified, and the expression of cell cycle genes related to proliferation was significantly upregulated in EBV-positive NPC cells, suggesting that B cells and cell cycle gene expression may be potential treatment targets. In addition, LAG-3 and HAVCR-2 were the most expressed checkpoint genes in CD8 + T cells and may thus be potential new checkpoint inhibition targets for NPC immunotherapy.

## Conclusions

High-throughput scRNA-seq technology is transforming biomedical research by establishing the transcriptome profiles of individual cells from different tissue samples [55, 153]. While there are various sequencing platforms and tools available for analyzing scRNA-seq data, each has its limitations. With ongoing technological developments, scRNA-seq will become more high-throughput, the cost of scRNA-seq and associated data analysis will decrease, the time required will be shorter, and various indicators such as accuracy and sensitivity will improve.

In time, scRNA-seq will be more widely used in the study of various tumor types, helping to develop better diagnostic and prognostic biomarkers, and design more accurate anticancer therapy to improve therapeutic efficacy and avoid tumor drug resistance. While the transcriptome of cells can be studied through scRNA-seq, there are some limitations to this method, particularly with regard to genomic and protein-level information. When CITE-seq was used in combination with scRNA-seq, the transcripts and surface marker proteins of cells could be detected simultaneously, and the phenotypes that could not be detected by scRNA-seq alone could be found [154]. The combination of scRNA-seq with genotyping can more accurately distinguish malignant cells from normal cells [101]. In the future, different omics technologies could combine with scRNA-seq technology to more comprehensively characterize individual cells. With deepening understanding of the cellular dynamics of cancer, the efficacy of personalized medicine will improve, ultimately saving lives and reducing the global burden of cancer on healthcare systems.

## Supplementary Information

**Additional file 1 **: **Fig. 1**. Application of scRNA-seq in tumor research. ScRNA-seq can be used to analyze the proportion of various immune cell types and immune repertoire characteristics, guiding choices related to immunotherapy. The analysis of single tumor cells can improve our understanding of drug resistance, invasion, and metastasis mechanisms, as well as cell origin and evolution, in turn aiding the choice of targeted drugs. T, T cell; B, B cell; NK, natural killer cell; TAM, tumor-associated macrophage; Th, helper T cell; Tc, cytotoxic T cell; Treg, regulatory T cell; Tm, memory T cell; Ts, suppressor T cell; B1, T cell independent B cell; B2, T cell dependent B cell.

**Additional file 2 **: **Table 1**. Overview of related studies using scRNA-seq.

**Additional file 3 **: **Table 2**. Overview of related articles using scRNA-seq.

**Additional file 4 **: **Table 3**. Overview of related studies using scRNA-seq.

**Additional file 5 **: **Table 4**. Overview of related articles using scRNA-seq.

**Additional file 6 **: **Table 5**. Overview of related articles using scRNA-seq.

**Additional file 7 **: **Table 6**. Overview of related articles using scRNA-seq.

**Additional file 8 **: **Table 7**. Overview of related articles using scRNA-seq.

## Data Availability

Not applicable.

## Abbreviations

scRNA-seq: Single-cell RNA sequencing
scLVM: Single-cell latent variable model
OT: Optimal transport
DE: Differentially expressed
GSE: Gene set enrichment
GRN: Gene regulatory network
GBM: Glioblastoma
HGG: High-grade glioma
LC: Lung cancer
SCLC: Small-cell lung carcinoma
NSCLC: Non-small-cell lung carcinoma
TME: Tumor microenvironment
AML: Acute myeloid leukemia
CTC: Circulating tumor cell
p-EMT: Partial epithelial-to-mesenchymal transition
TNBC: Triple negative breast cancer
MM: Multiple myeloma
cPC: Circulating plasma cell
EMP: Extramedullary plasmacytoma
PDX: Patient-derived xenograft
LUAD: Lung adenocarcinoma
MB: Medulloblastoma
SHH: Sonic Hedgehog
HCC: Hepatocellular carcinoma
TAM: Tumor-associated macrophage
CRC: Colorectal cancer
HCMB: Human cytomegalovirus
G34R/V: Glycine 34 to arginine or valine
RCC: Renal cell carcinoma
MRCC: Metastatic renal cell carcinoma
MNB: Multilayer network biomarker
OPC: Oligodendrocyte precursor cell
NPC: Nasopharyngeal carcinoma
EGFR: Epidermal growth factor receptor
