# Supplementary material for: What are the applications of single-cell RNA sequencing in cancer research: a systematic review
Source: J Exp Clin Cancer Res. 2021 May 11;40:163. doi: 10.1186/s13046-021-01955-1 (PMC8111731; doi:10.1186/s13046-021-01955-1)
Supplement: Supplementary file 1 — Additional file 1 : Fig. 1. Application of scRNA-seq in tumor research. ScRNA-seq can be used to analyze the proportion of various immune cell types and immune repertoire characteristics, guiding choices related to immunotherapy. The analysis of single tumor cells can improve our understanding of drug resistance, invasion, and metastasis mechanisms, as well as cell origin and evolution, in turn aiding the choice of targeted drugs. T, T cell; B, B cell; NK, natural killer cell; TAM, tumor-associated macrophage; Th, helper T cell; Tc, cytotoxic T cell; Treg, regulatory T cell; Tm, memory T cell; Ts, suppressor T cell; B1, T cell independent B cell; B2, T cell dependent B cell. [file 13046_2021_1955_MOESM1_ESM.pdf]

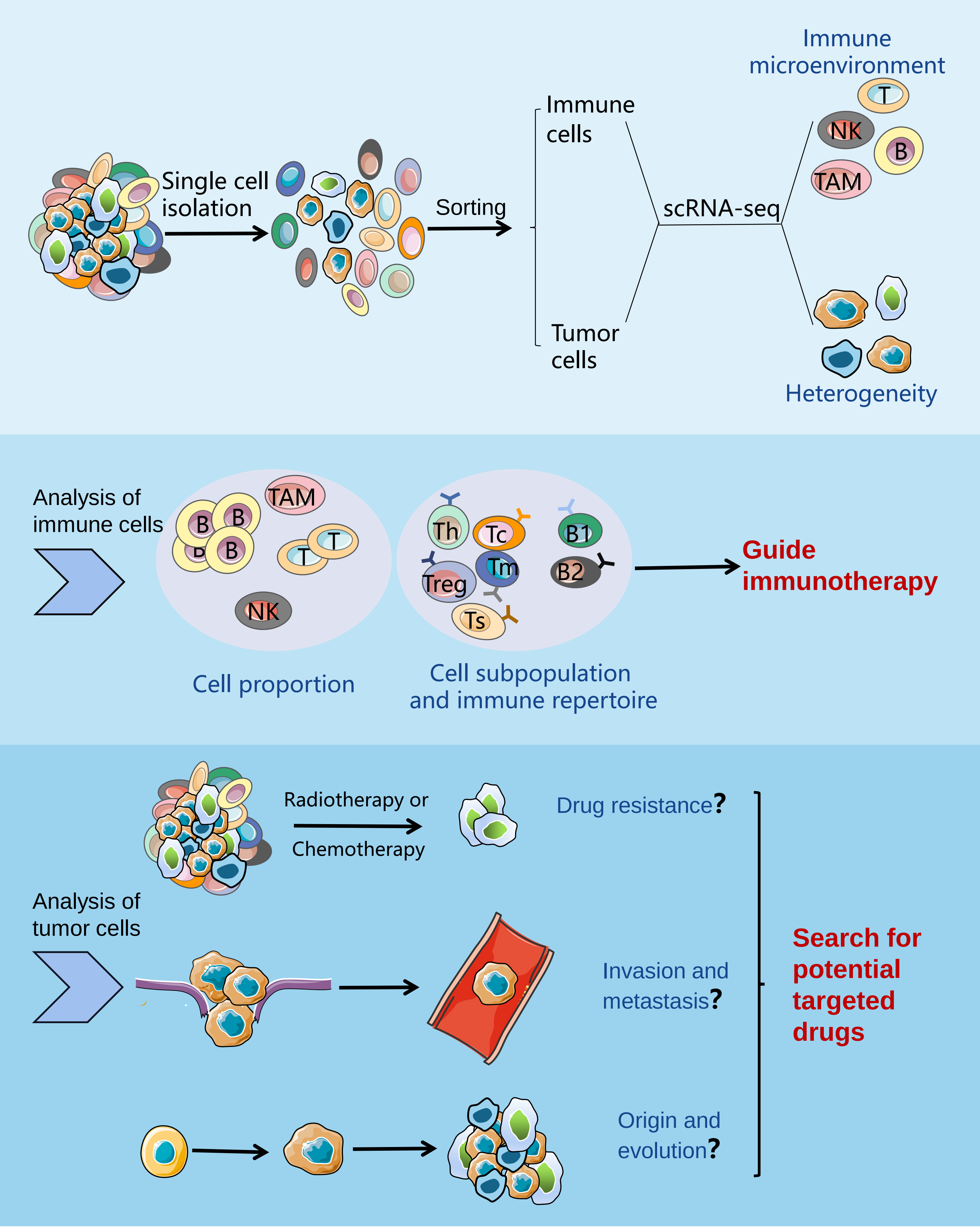
Single cell isolation
Sorting
Immune cells
Tumor cells
scRNA-seq
Immune microenvironment
T
NK
B
TAM
Heterogeneity
Analysis of immune cells
B
TAM
T
NK
Th
Tc
B1
Tm
B2
Treg
Ts
Cell proportion
Cell subpopulation and immune repertoire
Guide immunotherapy
Analysis of tumor cells
Radiotherapy or
Chemotherapy
Drug resistance?
Invasion and metastasis?
Origin and evolution?
Search for potential targeted drugs
