# Supplementary material for: What are the applications of single-cell RNA sequencing in cancer research: a systematic review
Source: J Exp Clin Cancer Res. 2021 May 11;40:163. doi: 10.1186/s13046-021-01955-1 (PMC8111731; doi:10.1186/s13046-021-01955-1)
Supplement: Supplementary file 2 — Additional file 2 : Table 1. Overview of related studies using scRNA-seq. [file 13046_2021_1955_MOESM2_ESM.pdf]

Table 1. Overview of related studies using scRNA-seq

Cancer types	Year	Analyzed cell types	Number of patients/cells	Technique	References
IDH1/2 wild-type primary GBMs	2014	Tumor cells	5; 430	scRNA-seq	[92]
Melanoma	2017	Tumor cells	3; 307	scRNA-seq	[95]
HNSCC	2017	Stromal, immune, and malignant cells	18; ~6000	scRNA-seq	[9]
HGG	2018	Tumor cells	8; ~24000	scRNA-seq	[93]
NSCLC	2018	Stromal cells of non-malignant lungs and lung tumors	8; 92948	scRNA-seq	[100]
AML	2019	Tumor cells and normal cells	21; 38410	combined scRNA-seq with single-cell genotyping	[101]
