# Supplementary material for: What are the applications of single-cell RNA sequencing in cancer research: a systematic review
Source: J Exp Clin Cancer Res. 2021 May 11;40:163. doi: 10.1186/s13046-021-01955-1 (PMC8111731; doi:10.1186/s13046-021-01955-1)
Supplement: Supplementary file 3 — Additional file 3 : Table 2. Overview of related articles using scRNA-seq. [file 13046_2021_1955_MOESM3_ESM.pdf]

Table 2. Overview of related articles using scRNA-seq

Cancer types	Year	Analyzed cell types	Number of patients/cells	Technique	References
Pancreatic cancer	2014	CTCs	Mice	scRNA-seq	[107]
HNSCC	2017	Stromal, immune, and malignant cells	18; ~6000	scRNA-seq	[9]
Breast cancer	2017	Tumor and immune cells	11; 515	scRNA-seq	[110]
MM	2019	Bone marrow myeloma cells and cPCs	21;	scRNA-seq	[112]
