# Supplementary material for: What are the applications of single-cell RNA sequencing in cancer research: a systematic review
Source: J Exp Clin Cancer Res. 2021 May 11;40:163. doi: 10.1186/s13046-021-01955-1 (PMC8111731; doi:10.1186/s13046-021-01955-1)
Supplement: Supplementary file 4 — Additional file 4 : Table 3. Overview of related studies using scRNA-seq. [file 13046_2021_1955_MOESM4_ESM.pdf]

Table 3. Overview of related studies using scRNA-seq

Cancer types	Year	Analyzed cell types	Number of patients/cells	Technique	References
Breast cancer	2014	Metastatic breast cancer cells	Three groups of breast cancer cell lines	scRNA-seq	[113]
LUAD	2015	Tumor cells	1; 34 (PDX)	scRNA-seq	[114]
Melanoma	2016	Malignant, immune, stromal and endothelial cells	19; 4645	scRNA-seq	[4]
Glioblastoma multiforme	2018	Tumor cells	1; over 200	scRNA-seq	[115]
MB	2019	Stromal and tumor cells	Mice	scRNA-seq	[118]
