# Supplementary material for: What are the applications of single-cell RNA sequencing in cancer research: a systematic review
Source: J Exp Clin Cancer Res. 2021 May 11;40:163. doi: 10.1186/s13046-021-01955-1 (PMC8111731; doi:10.1186/s13046-021-01955-1)
Supplement: Supplementary file 5 — Additional file 5 : Table 4. Overview of related articles using scRNA-seq. [file 13046_2021_1955_MOESM5_ESM.pdf]

Table 4. Overview of related articles using scRNA-seq

Cancer types	Year	Analyzed cell types	Number of patients/cells	Technique	References
HCC	2017	T cells in peripheral blood, tumor, and adjacent normal tissues	6; 5063	scRNA-seq	[8]
Glioma	2017	TAMs	19; 5455 (1274 published cells and 4181 novel cells)	scRNA-seq combined with public data	[130]
LUAD	2017	Immune cells in tumor, non-involved lung and blood cells	28; over 1800 cells	scRNA-seq	[131]
Breast cancer	2018	Immune cells from breast carcinomas, as well as matched normal breast tissue, blood, and lymph node	11; 72000	scRNA-seq	[132]
CRC	2018	T cells	12; 11138	scRNA-seq	[134]
