# Supplementary material for: What are the applications of single-cell RNA sequencing in cancer research: a systematic review
Source: J Exp Clin Cancer Res. 2021 May 11;40:163. doi: 10.1186/s13046-021-01955-1 (PMC8111731; doi:10.1186/s13046-021-01955-1)
Supplement: Supplementary file 6 — Additional file 6 : Table 5. Overview of related articles using scRNA-seq. [file 13046_2021_1955_MOESM6_ESM.pdf]

Table 5. Overview of related articles using scRNA-seq

Cancer types	Year	Analyzed cell types	Number of patients/cells	Technique	References
IDH1 or IDH2 mutant human oligodendrogliomas	2016	Tumor cells	6; 4347	scRNA-seq	[136]
IDH-O/IDH-A	2017	Tumor cells and TME	16; 14226	scRNA-seq	[137]
MB	2019	WNT, SHH, Group 3 and Group 4 tumors	36 (11 PDX models); 86890 (4181 novel cells and 78,156 published cells from mice)	scRNA-seq combined with public data	[139]
GBM	2019	Tumor cells	24; 8366	analyzed public scRNA-seq data	[138]
Glioma	2019	Glial progenitors and	Mice	scRNA-seq	[140]

		malignant cells			
Histone H3.3 G34R/V HGGs	2020	Tumor cells and normal cells	17; 64943	scRNA-seq	[141]
