# Supplementary material for: What are the applications of single-cell RNA sequencing in cancer research: a systematic review
Source: J Exp Clin Cancer Res. 2021 May 11;40:163. doi: 10.1186/s13046-021-01955-1 (PMC8111731; doi:10.1186/s13046-021-01955-1)
Supplement: Supplementary file 7 — Additional file 7 : Table 6. Overview of related articles using scRNA-seq. [file 13046_2021_1955_MOESM7_ESM.pdf]

Table 6. Overview of related articles using scRNA-seq

Cancer types	Year	Analyzed cell types	Number of patients/cells	Technique	References
RCC	2016	Tumor cells	1; 116 (82 PDX cells)	scRNA-seq	[143]
IDH-mutant astrocytoma	2019	Cancer cells and TME	;6341	Based on scRNA-seq data downloaded from GEO database	[144]
