# Supplementary material for: What are the applications of single-cell RNA sequencing in cancer research: a systematic review
Source: J Exp Clin Cancer Res. 2021 May 11;40:163. doi: 10.1186/s13046-021-01955-1 (PMC8111731; doi:10.1186/s13046-021-01955-1)
Supplement: Supplementary file 8 — Additional file 8 : Table 7. Overview of related articles using scRNA-seq. [file 13046_2021_1955_MOESM8_ESM.pdf]

Table 7. Overview of related articles using scRNA-seq

Cancer types	Year	Analyzed cell types	Number of patients/cells	Technique	References
GBM	2017	Tumor core and surrounding peripheral tissue	4; 3589	scRNA-seq	[145]
H3K27m-glioma	2018	Tumor cells	6; 3321	scRNA-seq	[146]
GBM	2019	Tumor cells	; 16128	scRNA-seq	[147]
NPC	2020	Tumor and immune cells	3; 2998	scRNA-seq	[7]
